# A Comparison of the Recruitment Success of Introduced and Native Species Under Natural Conditions

**DOI:** 10.1371/journal.pone.0072509

**Published:** 2013-08-08

**Authors:** Habacuc Flores-Moreno, Angela T. Moles

**Affiliations:** Evolution & Ecology Research Centre, School of Biological, Earth and Environmental Sciences, the University of New South Wales, Sydney, New South Wales, Australia; University Copenhagen, Denmark

## Abstract

It is commonly accepted that introduced species have recruitment advantages over native species. However, this idea has not been widely tested, and those studies that have compared survival of introduced and native species have produced mixed results. We compiled data from the literature on survival through germination (seed to seedling survival), early seedling survival (survival through one week from seedling emergence) and survival to adulthood (survival from germination to first reproduction) under natural conditions for 285 native and 63 introduced species. Contrary to expectations, we found that introduced and native species do not significantly differ in survival through germination, early seedling survival, or survival from germination to first reproduction. These comparisons remained non-significant after accounting for seed mass, longevity and when including a random effect for site. Results remained consistent after excluding naturalized species from the introduced species data set, after performing phylogenetic independent contrasts, and after accounting for the effect of life form (woody/non-woody). Although introduced species sometimes do have advantages over native species (for example, through enemy release, or greater phenotypic plasticity), our findings suggest that the overall advantage conferred by these factors is either counterbalanced by advantages of native species (such as superior adaptation to local conditions) or is simply too small to be detected at a broad scale.

## Introduction

Seeds and seedlings are exposed to many risks during establishment, such as predation, loss of viability in the soil, drought, herbivory, pathogen attack, shading, nutrient deprivation and competition [1]. As a result, seeds and seedlings experience the highest mortality rates of any life history stage [2]. A widely accepted hypothesis in invasion ecology is that introduced or invasive species have higher survival through the early stages of establishment than do native or non-invasive species [3–5]. Introduced species are often associated with dominance, rapid spread and fast population growth rates [6], and it is feasible that higher survival of introduced species through the early stages of establishment could contribute to these.

The idea that introduced species might have higher recruitment success is based on both theoretical arguments and empirical observations of three main mechanisms: enemy release, higher plasticity, and faster growth rates. Enemy release could allow introduced species to escape from specialist seed predators and herbivores in their novel range [7]. However, evidence for enemy release has been inconsistent [8–10]. Greater plasticity of introduced species could allow them to germinate under a wider range of environmental conditions and promote their higher tolerance to environmental stress in the early stages of life. However, evidence for introduced species’ greater plasticity shows conflicting results [11–14]. Faster growth rates could shorten the time introduced species spend in early vulnerable stages of the life cycle and/or reduce the time to reproduction, thus reducing a species’ exposure to mortality [4,15]. However, the available data for this is also varied [11,16–18]. That is, although some theories and data suggest that introduced species should have advantages over native species, the evidence has been inconsistent. Moreover, the magnitude of the positive effect of these mechanisms on introduced species’ fitness, specifically in the form of survival through the different stages of recruitment, is unclear. Our main objective is to provide a large scale test of the idea that introduced species have superior recruitment success than do native species.

It has been suggested that introduced species germinate faster, to a higher percentage and in a wider range of conditions than do native species [5] and that high germination success promotes invasiveness [3]. Empirical studies have found higher germination percentages in introduced or invasive species [19–24], lower germination in introduced or invasive species [25–27], mixed results [28–31] and non-significant differences in germination between introduced or invasive and native or non-invasive species [32–34]. These studies used a wide variety of approaches, but broadly can be divided into studies performed under natural conditions without manipulation, and studies performed under experimental or artificial conditions (e.g. in greenhouses, with supplementary watering, or herbivore exclosures). The latter have described the proportion of germination across an amazing range of species under experimental or artificial conditions (e.g. [20,21,34]). Conversely, studies performed under natural conditions without manipulation have been commonly limited to few subject species (e.g. [19,23,31]). Thus, many studies have compared germination of introduced and native species; however nobody has ever compared the survival through germination of introduced and native species under natural conditions on a broad scale. This is our first aim.

Pyšek and Richardson [3] proposed that higher rates of seedling survival and establishment should promote invasiveness in introduced species. However these authors highlight the lack of large comparative datasets available to test this idea. Some evidence shows that introduced or invasive species have higher seedling survival than natives [35,36]. However, several studies have found non-significant differences in seedling survival between native and introduced or invasive species [19,37–40]. Others have found that native species have higher seedling survival than do invasive species [23,41], while some have found mixed results [28,42,43]. One possible explanation for the abundance of mixed results is that the majority of these studies focus on small numbers of species, usually from one region. Furthermore, many studies compare seedling survival and/or establishment of introduced and native species in greenhouse, laboratory conditions, or garden experiments where herbivory, water/light-stress and competition are often controlled for. Although studies under these conditions tell us what introduced species are capable of doing compared to native species, everything else being equal, they do not tell us about how introduced species are dealing with natural environmental conditions, selective pressures and interactions. Thus, our second aim is to compare the early seedling survival of introduced and native species at a broad scale under natural conditions.

Seed mass is an ecologically important trait that affects the recruitment strategy of plants [1]. Plants with bigger seeds have higher early seedling survival than do those with smaller seeds [44]. Thus, it is possible that a difference in seed mass between native and introduced species could mask or generate differences in early seedling survival between introduced and native species. Therefore our third aim is to assess whether introduced and native species differ in early seedling survival under natural conditions, once the effect of seed mass had been accounted for. No significant relationship has been found between seed mass and proportion of germination, or between seed mass and survival from seedling to reproduction [44], and the advantages of large seed size are known to be restricted to early establishment –usually no later than cotyledon phase [45]. Therefore, we did not control for seed mass in our assessment of differences in germination or survival from seed to fruiting.

If introduced species do not survive until reproduction, then no naturalized or introduced population can succeed. Previous studies comparing survival from germination to first reproduction between introduced and native species have produced contrasting results, including, higher survival in introduced or invasive species [46], mixed results [47,48] and lower survival of invasive species’ in their introduced range [46,47,49,50]. However, all previous work has been on pairs or small groups of species. The fourth aim of our study is to ask whether introduced species have higher survival from germination to first reproduction than do native species at a broad scale, under natural conditions.

It has been proposed that short life cycles will be favoured among introduced species [4,51]. If this is the case then differences in longevity between introduced and native species could overshadow differences in recruitment by affecting the time over which species are exposed to mortality. Our fifth aim is to assess whether introduced and native species differ in survival through germination, early seedling survival, and survival from germination to first reproduction once the effect of longevity has been taken into account. For the comparison of early seedling survival between introduced and native species we first accounted for the effect of longevity by itself; then we ran a model that also included a term for seed mass.

In summary, the hypotheses that we addressed in this paper were:

1. Introduced species have higher survival through germination (seed to seedling survival) than do native species.
2. Introduced species have a higher early seedling survival (survival for the first week after germination) than do native species.
3. Introduced species have higher early seedling survival than do native species once seed mass has been accounted for.
4. Introduced species have higher survival from germination to first reproduction than do native species.
5. Introduced species have higher recruitment success than do native species once the effect of longevity has been accounted for.

## Methods

### Ethical statement

No permission or approval was required for obtaining the data included in this study because all the data were extracted from published sources.

We began with the database for germination, seedling survival and seedling survival from germination to first reproduction generated by Moles et al. [44]. This database contains studies published up to week 38, 2002 with information on germination and seedling survival. Therefore, we searched ISI web of knowledge for papers in English from week 38, 2002 to week 3, 2012 containing the words ‘seedling survival’ and ‘germination’ restricted by the terms ‘weed$’, ‘introduced’, ‘invasive’, ‘non-invasive’, ‘naturali*’, ‘alien’, ‘non-native’, and ‘noxious’ in order to obtain more data points for introduced species’ germination, seedling survival, and survival from germination to first reproduction.

Only studies measuring the survival of seeds and seedlings of both native and introduced species in natural conditions were included. Studies were excluded if seedling were raised in pots, within exclosures, under shelters, with extra watering, with pesticides, with weeding, or with supplementary fertilization. Studies were also excluded if individual seedling survival was not followed from the day of emergence or if they were transplanted after emergence. Only studies with a minimum sample size of ten individuals were included. In total our database contained information for 348 species from 90 families, from 186 different sites around the world. This included data for 285 native species and 63 introduced species. Of the 63 introduced species in our database, two were classified as naturalized and 61 as introduced.

We extracted information on survival through germination (seed to seedling survival), early seedling survival (survival through one week from seedling emergence), survival from germination to first reproduction, seed mass and introduced/native status. These data were extracted from three sources from the papers in our database, with the following order of preference: 1) tables, 2) text and 3) graphs (using Datathief III [52]). Additional data on introduced/native status were compiled from the global compendium of weeds [53] and environmental agencies from the regions where the studies took place. Additional seed mass data were compiled from Moles et al. [54] and Kew botanical gardens’ seed information database [55].

Ideally, longevity should be measured as a continuous variable. Unfortunately, continuous longevity data are very scarce. We collected categorical longevity data (annual, biennial and perennial; lifespan categories) for 345 species, and continuous maximum recorded longevity data (continuous longevity) for 128 species. Lifespan categories data were collected in decreasing order of preference from 1) the same papers as survival data, 2) environmental agencies from the region in which the study took place or 3) papers found in main references. Continuous longevity data were collected from the global literature (Table S6). We have lifespan categories data for 99.7% of our species, and continuous longevity data for 37% of our species. That is, the strength of the lifespan categories data is coverage, while the strength of the continuous longevity data is resolution.

Before analysis we log_10_- transformed the seed mass and continuous longevity data, and logit-transformed data for survival through germination, early seedling survival, and survival from germination to first reproduction [56]. To avoid problems with species with survival values equal to 0 or 1 (0 or 100% survival respectively) we added or subtracted the smallest non-zero value within each of the recruitment stages to these species [56]. Species recorded both in a native and introduced region were statistically weighted such that each species had a total statistical weight of one.

To determine whether introduced species have higher survival through germination, early seedling survival (survival for the first week after germination) and survival from germination to first reproduction than do native species, we ran *t*-tests assuming unequal variance with species’ status (introduced/native) as our predictor variable and survival through germination, early seedling survival, or survival from germination to first reproduction as our dependent variables.

To determine whether introduced species have higher early seedling survival than do native species once the effect of seed mass has been accounted for, we used a linear model where the predictor variables were species’ status (introduced/native) and seed mass, and the dependent variable was early seedling survival.

To determine whether introduced and native species differ in survival through germination, early seedling survival, and survival from germination to first reproduction after accounting for the effect of longevity we ran a linear model where our predictor variables were species’ status (introduced/native) and lifespan categories (annual, biennial and perennial life cycles), and our dependent variables were survival through germination, early seedling survival and seedling survival to first reproduction. For early seedling survival, we also ran a linear model that accounted for the effect of species’ status, seed mass, lifespan categories and their interactions. In this linear model the predictor variables were species’ status (introduced/native), seed mass and lifespan categories (annual, biennial and perennial life cycles) and the dependent variable was early seedling survival. The low number of biennial species with data for early seedling survival (n = 7), and survival from germination to first reproduction (n = 5) affected the number of degrees of freedom needed for these linear models. In our study annual, biennial and perennial plants did not significantly differ in early seedling survival or survival from germination to first reproduction. In order to have a statistically more powerful comparison we merged the biennial and perennial species into one lifespan category. These results do not qualitatively differ from comparisons where biennial plants have been excluded (Table S1). Finally, we compared the continuous longevity of introduced and native species using a *t*-test and re-ran all longevity analyses using continuous longevity data (Table S6). All analyses were performed in R 2.15.1 [57].

### Data considerations

In our main analyses, we asked whether differences in seed mass and longevity between native and introduced species might have affected our results. Here, we consider some additional factors: site to site variation, degree of invasiveness of the study species, phylogeny, and life form (woody/non-woody).

#### 1. Site to site variation

Some data points in our database come from the same site. To explicitly account for their non-independence we ran all analyses using mixed models including terms for: lifespan categories (annual, biennial and perennial), status (introduced/native), and a random term for site. The model for early seedling survival also included a term for seed mass because early seedling survival is affected by it ( [44]; see above).

#### 2. Naturalized and invasive introduced species

Not all introduced species are invasive. However of the 63 introduced species included in our study, two were classified as naturalised and 61 were classified as invasive (*sensu* [58]) by the primary studies or environmental agencies of the countries were the studies took place. Investigating the effect of species’ invasiveness on recruitment was beyond the scope of our study. However, we did ask whether our inclusion of non-invasive introduced species might have obscured a significant result.

#### 3. Phylogeny

Phylogenetic analyses investigate the evolution of traits. These analyses explicitly assume that the traits under comparison are heritable [59]. Species’ status (introduced/native) and survival are non-heritable ecological traits. Species do not evolve to be introduced (i.e. the change from native to introduced state does not evolve along a phylogeny) and while some traits associated with survival are heritable, survival also depends on non-heritable factors such as environmental conditions, intensity of interactions with herbivores, predators, pathogens and other plants, and chance. Thus, phylogenetic analyses are not appropriate for our data. Nevertheless, to dispel any doubts about the robustness of our results stemming from the potential non-independence of data points due to phylogenetic relatedness, we performed a phylogenetic independent contrast analysis (Table S3). First, we constructed a phylogeny of plant species included in our dataset using PHYLOMATIC ( [60,61]; PHYLOMATIC tree version R20100701), and generated phylogenetically independent contrasts using the Analysis of Traits module in PHYLOCOM 4.2 [62]. Finally, we used one-sample *t*-tests to determine whether changes in species’ status had been consistently associated with changes in survival rate through the evolutionary history of these species.

#### 4. Life-form (woody/non-woody)

We tested whether there was a difference in proportion of introduced and native species with woody or non-woody growth form using a Chi squared test. Then, we ran linear models where our predictor variables were species status (introduced/native) and life-form (woody/non-woody) and our dependent variables were survival through germination, early seedling survival, and survival from germination to first reproduction.

## Results

**Figure 1:**
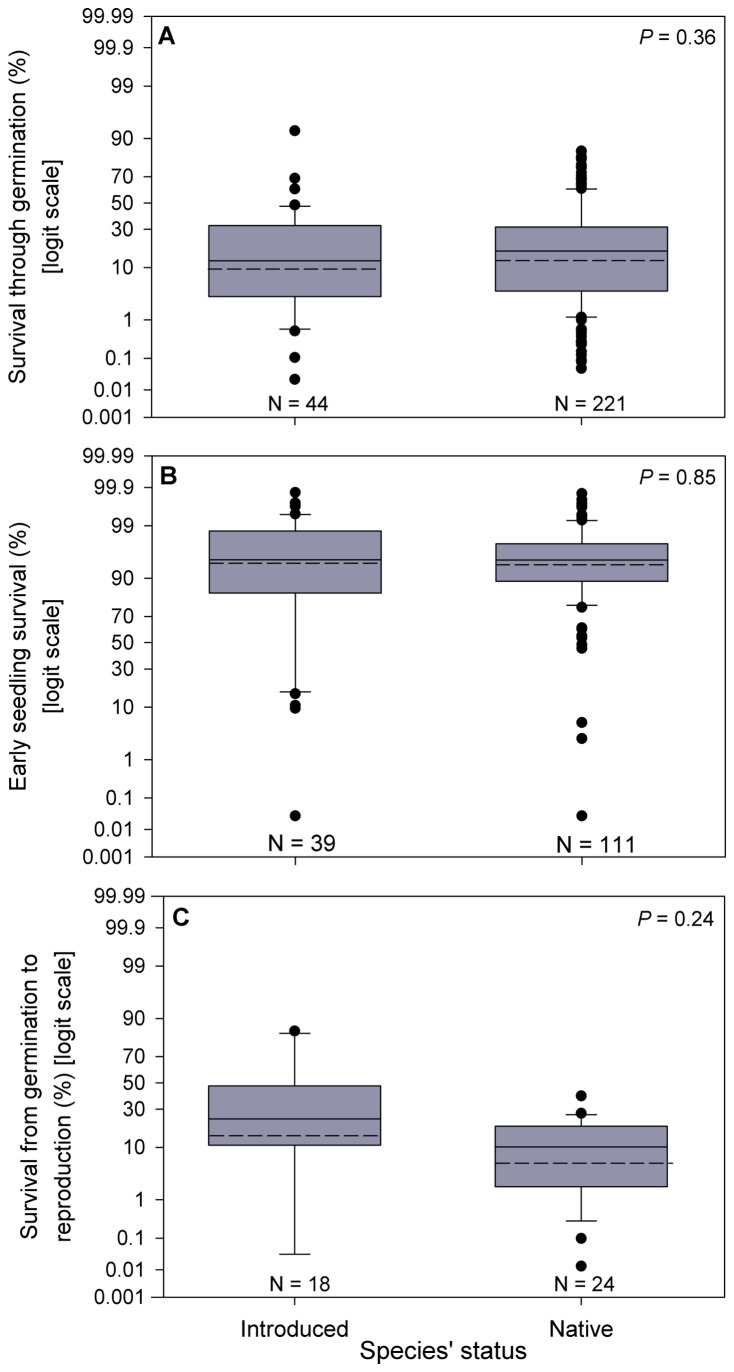
Comparison of introduced and native species recruitment success. Introduced and native species’ (A) survival through germination, (B) early seedling survival (one week survival after germination) and (C) survival from germination to first reproduction. Black dashed lines represent geometric mean values. Boxes represent the 25^th^, 50^th^ and 75^th^ percentiles. Whiskers represent the 10^th^ and 90^th^ percentiles, outliers are represented as points.

Contrary to expectations, we did not find differences in introduced and native species’ survival through germination (seed to seedling survival; *P* = 0.36; Figure 1A), early seedling survival (one week survival from seedling emergence; *P* = 0.85; Figure 1B), or survival from germination to first reproduction (*P* = 0.22; Figure 1C). On average, 12% of the seeds that enter germination survive to seedling, 94% of the individuals that enter the seedling stage survive for one week and only 8% of seedlings survive from germination to first reproduction.

After controlling for lifespan categories, the differences between introduced and native species’ survival through germination (*P* = 0.34; Table S2), early seedling survival (*P* = 0.84; Table S2) and survival from germination to first reproduction (*P* = 0.75; Table S2) remained non-significant. Lifespan categories explained substantially more variation in survival through germination (2.7-fold), early seedling survival (3-fold), and survival from germination to first reproduction (9-fold) than did species’ status. When we compared the continuous longevity of introduced and native species we found that, on average, introduced species had significantly shorter lifespans (~9.6 years, *P* = 0.0005) than did native species (~53.6 years; Table S6). However, we still did not find a significant difference on survival through germination (*P* = 0.07), early seedling survival (*P* = 0.26), or survival from germination to first reproduction (*P* = 0.65) between introduced and native species (Table S6).

We did not find a significant difference between native and introduced species’ early seedling survival after controlling for seed mass (*P* = 0.49; Table S1). When we compared the early seedling survival of native and introduced species once the effect of lifespan categories, seed mass and their interactions had been accounted for, we did not find a significant effect of species’ status on early seedling survival (*P* = 0.27; Table S2). However, we found a significant positive effect of lifespan categories (*P* = 0.002, Table S2). Lifespan categories explained far more variation (*R* ^2^ = 0.07) than did either species’ status (*R* ^2^ = 0.008) or the interaction between species’ status and lifespan categories (*R* ^2^ = 0.01). Results remained qualitatively similar when using continuous longevity instead of lifespan categories (Table S6).

### Data considerations

#### 1. Site to site variation

Analyses including a random effect for site were broadly consistent with previous results, showing that species’ status does not have a significant effect on survival through germination (*P* = 0.18; Table S3), early seedling survival (*P* = 0.48; Table S3), or survival from germination to first reproduction (*P* = 0.79; Table S3). Overall, site accounted for 31%, 40% and 6% of the variation in survival through germination, early seedling survival and seedling survival to reproduction respectively. This is in line with the results of previous broad scale data compilations, where site variation has typically explained about half of the observed variation (e.g. [63–65]).

#### 2. Naturalized and invasive introduced species

After excluding the two naturalized species from our database the difference between introduced and native species’ survival through germination, early seedling survival, and survival from germination to first reproduction remained non-significant (*P* = 0.28, 0.59 and 0.22, respectively).

#### 3. Phylogeny

Like our cross-species analyses, phylogenetic analyses showed no significant difference between introduced and native species’ survival through germination (*P* = 0.91; Table S4), early seedling survival (*P* = 0.32; Table S4), or survival from germination to first reproduction (*P* = 0.65; Table S4).

#### 4. Life-form (woody/non-woody)

We found that native species are significantly more likely to be woody (Χ^2^ = 12.92, d.f. = 1, *P* = 0.0003; Table S5). However, we found no significant effect of species’ status on survival through germination (*P* = 0.47; Table S5), early seedling survival (*P* = 0.72; Table S5), and survival from germination to first reproduction (*P* = 0.48; Table S5) once the effect of life-form (woody/non-woody) was accounted for. Only in the case of early seedling survival was there a significant effect of life form (*P* = 0.02; Table S5).

## Discussion

Our most important finding is that introduced and native species do not significantly differ in survival through germination, early seedling survival, and survival from germination to first reproduction. These results remained non-significant after accounting for seed mass, longevity, when including a random effect for site, excluding non-invasive introduced species, accounting for phylogeny, or accounting for life form. These findings were contrary to our expectations, and also to expectations in the literature [3,5,20,21,34]. Our results show that the idea that germination success, and seedling survival and establishment are key drivers of introduced species’ spread and dominance in new environments needs to be seriously reconsidered. After all, given that the native species have had generations of selection for traits that favour survival under the local conditions we may have expected native species to exceed, or at least match, the survival of introduced species.

Several studies have proposed that introduced species could benefit from enemy release, faster growth rate or phenotypic plasticity. Our data suggest that any advantage introduced species accrue as a result of these factors is either small enough in magnitude or uncommon enough that it does not make a detectable difference to overall survival. One possibility is that the advantages of introduced species are balanced by the superior adaptation of natives to their environment. Our results are consistent with recent meta-analyses that have found that enemy release [8,9] and plasticity in invasive and/or introduced species do not always result in higher fitness or performance [12]. In the case of plasticity, our result could also be consistent with introduced species’ not having higher plasticity in the first place (see 13, for a more detailed discussion see 14,66). Evidence for higher relative growth rates is mixed [11,16–18], and no clear relationship between relative growth rate and survival to adulthood has yet been described.

Many studies have shown significant differences in functional or morphological traits of introduced and native species (e.g. [3,4,11,16,18]). These traits are often chosen for study because they are thought to be indicators of important processes such as seed production, seed dispersal, recruitment and growth. However, our study shows no link between species’ status (introduced or native) and higher success through recruitment. This discrepancy between the findings of trait-based studies (e.g. [16,18]) and process-based studies (e.g. [67]) merits further investigation. That is, invasion ecology needs to rigorously test the relationship between traits proposed to be related to introduced/invasive species and introduced/invasive species’ high performance, specifically in the form of higher survival, higher fecundity and higher competitive ability.

Our findings also carry an important message for managers – we must not assume that all introduced species are super-plants. Although some introduced species are clearly extremely successful in their new ranges (e.g. *Lythrum salicaria* has high recruitment success in North America [68], as does *Berberis* *darwinii* in New Zealand [69]), both introduced and native species have a wide range of recruitment success, and on average introduced species’ survival is no better than that of native species.

If introduced species have no advantage during recruitment, then it is worth asking where in the life cycle they do out-perform native species. Theory suggests that introduced species, particularly invasive ones, will have advantages over native species in the form of abundant seed output [3], superior seed dispersal [70,71], faster and higher proportions of germination under a wider range of environmental conditions [5] and higher seedling survival and establishment [3]. Many empirical studies show that introduced species germinate faster [20–23,33,34] and under a wider range of conditions [24,28] than their native counterparts. However, the evidence does not always support this view [29,30],, and even if introduced species do have faster germination under wider range of conditions, these advantages do not translate to higher survival through the early stages of recruitment (Figure 1). Introduced species produce as much as 6.7 times more seeds per plant per year than do native species [72], but a compilation of published data found no clear difference in seed dispersal distance between native and introduced species [67]. That is, higher seed production might be the only life history stage where introduced species have a general advantage over native species. However not all of the seeds plants produce are viable [73–75]. Another important topic for future research is to determine whether introduced species produce a higher proportion of viable seeds than do native species.

Ramula et al. [76] showed that introduced species’ population growth rate is on average 9.4 times higher compared to that of native species. This is roughly consistent with the 6.7 times higher seed production [72] being the only detectable advantage of introduced species. However, Ramula et al. [76] also highlighted the tendency for ecologists to measure population growth rates of introduced species during the exponential growth phase, but to measure native species’ population growth rates when they are stable or declining. It is worth asking: when during the introduction process does the effect of higher seed production start, how long does it last and does seed production differ between introduced and native populations of the same species?

There are far fewer data available for survival from germination to first reproduction than for survival through germination or early seedling survival. This is most likely because following the fate of seeds beyond the early stages of survival particularly in long lived species is extremely difficult. In our study we found no significant difference between introduced (n = 18) and native species’ (n = 24) survival from germination to first reproduction. Given our modest sample size, a lack of statistical power might have contributed to the non-significant difference between introduced and native species’ survival from germination to first reproduction (Figure 1C). However, only 28 replicates would be needed to accurately (90% accuracy) detect a 5% difference between introduced and native species’ survival from germination to first reproduction (using a power test [77]). That is, any difference between introduced and native species’ survival from germination to first reproduction would have to be fairly small in order to not have been detected in our study.

In summary, we have shown that on average, introduced species do not have higher recruitment success than do native species. These results overturn our traditional understanding of recruitment as a key driver of introduced species success. To understand the driving factors behind introduced species’ success, we need to quantify the relevance of traits and mechanisms proposed to be of importance to introduced species under natural conditions. It is time for invasion ecology to go through a careful synthesis and integration of the theories and empirical information and to determine whether introduced species actually do have, or should even be expected to have, lasting advantages over native species.

## Supporting Information

Table S1 Comparison of introduced and native species’ early seedling survival and survival from germination to first reproduction, excluding biennial species.
(DOC)

Table S2 Comparison of introduced and native species’ recruitment success once the effect of lifespan categories and seed mass have been accounted for.
(DOC)

Table S3 Comparison of introduced and native species recruitment success after accounting for seed mass, lifespan categories, and when including a random effect for site.
(DOC)

Table S4 Phylogenetic independent contrast of introduced and native species recruitment success.
(DOC)

Table S5 Comparison of introduced and native species’ recruitment success once the effect of life form (woody/non-woody) has been taken into account.
(DOC)

Table S6 Comparison of introduced and native species’ recruitment success once the effect of continuous longevity has been accounted for.
(DOC)
