# Supplementary material for: A Comparison of the Recruitment Success of Introduced and Native Species Under Natural Conditions
Source: PLoS One. 2013 Aug 8;8(8):e72509. doi: 10.1371/journal.pone.0072509 (PMC3738575; doi:10.1371/journal.pone.0072509)
Supplement: Table S1 — Comparison of introduced and native species’ early seedling survival and survival from germination to first reproduction, excluding biennial species. (DOC) [file pone.0072509.s001.doc]

**Table S1:** Comparison of introduced and native species’ early seedling survival and seedling survival to reproduction excluding data for biennial species.

**1)** **EARLY SEEDLING SURVIVAL (ONE WEEK AFTER GERMINATION) AND LONGEVITY**

| **Term** | **Sum of squares** | **d.f.** | ***P*** |
| --- | --- | --- | --- |
| Intercept | 20.85 | 1 | 0.02 |
| Species' status | 10.42 | 1 | 0.10 |
| Lifespan categories | 29.39 | 1 | 0.006 |
| Species status × Lifespan categories | 13.84 | 1 | 0.06 |
| Residuals | 535.51 | 142 |  |

**2) SEEDLING SURVIVAL TO REPRODUCTION AND LONGEVITY**

| **Term** | **Sum of squares** | **d.f.** | ***P*** |
| --- | --- | --- | --- |
| Intercept | 33.17 | 1 | 0.03 |
| Species' status | 0.54 | 1 | 0.78 |
| Lifespan categories | 2.5 | 1 | 0.55 |
| Species status × Lifespan categories | 8.87 | 1 | 0.26 |
| Residuals | 232.31 | 34 |  |

**3) EARLY SEEDLING SURVIVAL (ONE WEEK AFTER GERMINATION), LONGEVITY AND SEED MASS**

| **Term** | **Sum of squares** | **d.f.** | ***P*** |
| --- | --- | --- | --- |
| Intercept | 13.5 | 1 | 0.07 |
| Species' status | 4.88 | 1 | 0.28 |
| Lifespan categories | 30.96 | 1 | 0.007 |
| Seed mass | 0.74 | 1 | 0.67 |
| Species status × Lifespan categories | 11.25 | 1 | 0.10 |
| Species status × Seed mass | 0.07 | 1 | 0.90 |
| Seed mass × Lifespan categories | 2.56 | 1 | 0.43 |
| Species status × Lifespan categories × Seed mass | 0.07 | 1 | 0.90 |
| Residuals | 456.39 | 111 |  |
