# Supplementary material for: A Comparison of the Recruitment Success of Introduced and Native Species Under Natural Conditions
Source: PLoS One. 2013 Aug 8;8(8):e72509. doi: 10.1371/journal.pone.0072509 (PMC3738575; doi:10.1371/journal.pone.0072509)
Supplement: Table S2 — Comparison of introduced and native species’ recruitment success once the effect of lifespan categories and seed mass have been accounted for. (DOC) [file pone.0072509.s002.doc]

**Table S2:** Comparison of introduced and native species’ recruitment success once the effect of lifespan categories and seed mass have been accounted for.

Longevity could affect the recruitment survival of introduced and native species. In order to control for this confounding factor we ran a linear model were our predictor variables where lifespan categories (annual, biennial and perennial), species’ status (introduced and native) and the interaction between lifespan categories and status, and our response variables were survival to germination, early seedling survival (one week survival after emergence) and survival from germination to first reproduction.

Next, to account for the effect of seed mass on early seedling survival we ran a linear model where the predictor variables were seed mass, species status and their interaction and the response variable was early seedling survival.

Finally we ran a linear model to account for the effect of seed mass, lifespan categories and their interaction on the early seedling survival of introduced and native species. In this case the predictor variables were seed mass, lifespan categories and their interactions and the response variable was early seedling survival.

All analyses were ran on logit-transform survival data and log-transform seed mass data.

**1) SURVIVAL THROUGH GERMINATION AND LIFESPAN CATEGORIES**

| **Term** | **Sum of squares** | **d.f.** | ***P*** |
| --- | --- | --- | --- |
| Intercept | 35.86 | 1 | 0.003 |
| Species’ status | 2.32 | 1 | 0.89 |
| Lifespan categories | 6.34 | 2 | 0.8 |
| Species’ status × Lifespan categories | 0.34 | 2 | 0.84 |
| Residuals | 1098.17 | 261 |  |

**2) EARLY SEEDLING SURVIVAL (ONE WEEK AFTER GERMINATION) AND LIFESPAN CATEGORIES**

| **Term** | **Sum of squares** | **d.f.** | ***P*** |
| --- | --- | --- | --- |
| Intercept | 20.1 | 1 | 0.03 |
| Species’ status | 8.74 | 1 | 0.14 |
| Lifespan categories | 26.73 | 2 | 0.04 |
| Species’ status × Lifespan categories | 13.64 | 2 | 0.19 |
| Residuals | 480.19 | 120 |  |

**3) SURVIVAL FROM GERMINATION TO FIRST REPRODUCTION AND LIFESPAN CATEGORIES**

| **Term** | **Sum of squares** | **d.f.** | ***P*** |
| --- | --- | --- | --- |
| Intercept | 33.17 | 1 | 0.03 |
| Species’ status | 0.54 | 1 | 0.77 |
| Lifespan categories | 4.9 | 2 | 0.69 |
| Species’ status × Lifespan categories | 8.93 | 2 | 0.51 |
| Residuals | 237.86 | 37 |  |

**4) EARLY SEEDLING SURVIVAL (ONE WEEK AFTER GERMINATION) AND SEED MASS**

| **Term** | **Sum of squares** | **d.f.** | ***P*** |
| --- | --- | --- | --- |
| Intercept | 284.11 | 1 | <.0001 |
| Species’ status | 1.98 | 1 | 0.49 |
| Seed mass | 13.47 | 1 | 0.07 |
| Species’ status × Seed mass | 2.12 | 1 | 0.47 |
| Residuals | 494.68 | 122 |  |

**5) EARLY SEEDLING SURVIVAL (ONE WEEK AFTER GERMINATION), SEED MASS, AND LIFESPAN CATEGORIES**

| **Term** | **Sum of squares** | **d.f.** | ***P*** |
| --- | --- | --- | --- |
| Intercept | 13.5 | 1 | 0.07 |
| Species’ status | 4.88 | 1 | 0.27 |
| Longevity | 38.6 | 1 | 0.002 |
| Seed mass | 0.74 | 1 | 0.66 |
| Species status × Lifespan categories | 11.79 | 1 | 0.09 |
| Species status × Seed mass | 0.07 | 1 | 0.9 |
| Seed mass × Lifespan categories | 2.57 | 1 | 0.42 |
| Species’ status × Seed mass × Lifespan categories | 0.07 | 1 | 0.9 |
| Residuals | 512.86 | 130 |  |
