# Supplementary material for: A Comparison of the Recruitment Success of Introduced and Native Species Under Natural Conditions
Source: PLoS One. 2013 Aug 8;8(8):e72509. doi: 10.1371/journal.pone.0072509 (PMC3738575; doi:10.1371/journal.pone.0072509)
Supplement: Table S3 — Comparison of introduced and native species recruitment success after accounting for seed mass, lifespan categories, and when including a random effect for site. (DOC) [file pone.0072509.s003.doc]

**Table S3.** Comparison of introduced and native species recruitment success after accounting for seed mass, lifespan categories, and when including a random effect for site.

To test the influence of site to site variation on the recruitment of introduced and native species we ran a linear mixed model in the same dataset used for our linear model were our predictor variables were species’ status, lifespan categories and a random effect for site and or dependent variables were survival through germination, early seedling survival and survival from germination to first reproduction. In the case of the early seedling survival we also included a predictor variable for seed mass because the significant relationship found between these two traits (see main text; ).

All analyses were ran on logit-transform survival data and log-transform seed mass data.

**1) SURVIVAL THROUGH GERMINATION, LIFESPAN CATEGORIES, AND RANDOM EFFECT FOR SITE**

| **Term** | **Estimate** | ***P*** |
| --- | --- | --- |
| Intercept | -1.94 | < 0.01 |
| Species’ status | 0.75 | 0.18 |
| Lifespan categories (biennia)l | 0.56 | 0.64 |
| Lifespan categories (perennial) | -0.08 | 0.89 |
| Species status × Lifespan categories (biennial) | -0.8 | 0.87 |
| Species status × Lifespan categories (perennial) | -0.79 | 0.4 |

**2) EARLY SEEDLING SURVIVAL, SEEDMASS, LIFESPAN CATEGORIES, AND RANDOM EFFECT FOR SITE**

| **Term** | **Estimate** | ***P*** |
| --- | --- | --- |
| Intercept | 3.35 | < 0.01 |
| Species’ status | -0.26 | 0.47 |
| Lifespan categories (biennial/perennial) | 0.007 | 0.29 |
| Seed mass | -0.37 | 0.78 |
| Species’ status × Lifespan categories (biennial/perennial) | -1.01 | 0.13 |
| Species’ status × Seed mass | 0.54 | 0.87 |
| Lifespan categories (biennial/perennial) × Seed mass | 0.55 | 0.51 |
| Species’ status × Lifespan categories (biennial/perennial) × Seed mass | -0.28 | 0.96 |

**3) SURVIVAL FROM GERMINATION TO FIRST REPRODUCTION, LIFESPAN CATEGORIES, AND RANDOM EFFECT FOR SITE**

| **Term** | **Estimate** | ***P*** |
| --- | --- | --- |
| Intercept | -2.58 | 0.03 |
| Species’ status | 0.06 | 0.79 |
| Lifespan categories (biennial/perennial) | 0.69 | 0.49 |
| Species’ status × Lifespan categories (biennial/perennial) | -1.39 | 0.28 |

**References**

1. Moles AT, Westoby M (2004) Seedling survival and seed size: a synthesis of the literature. J Ecol 92: 372-383.
