# Supplementary material for: A Comparison of the Recruitment Success of Introduced and Native Species Under Natural Conditions
Source: PLoS One. 2013 Aug 8;8(8):e72509. doi: 10.1371/journal.pone.0072509 (PMC3738575; doi:10.1371/journal.pone.0072509)
Supplement: Table S4 — Phylogenetic independent contrast of introduced and native species recruitment success. (DOC) [file pone.0072509.s004.doc]

**Table S4:** Phylogenetic independent contrast of introduced and native species recruitment success.

| **Trait** | **number of contrasts** | **Mean** | **Standard deviation** | ***P*** |
| --- | --- | --- | --- | --- |
| Survival to germination (%) | 265 | -0.04 | 1.53 | 0.9 |
| Early seedling survival (%) | 150 | 0.51 | 2.56 | 0.31 |
| Survival from germination to first reproduction (%) | 42 | 0.43 | 3.12 | 0.65 |

All analyses were done on logit-transform survival data.
