# Supplementary material for: A Comparison of the Recruitment Success of Introduced and Native Species Under Natural Conditions
Source: PLoS One. 2013 Aug 8;8(8):e72509. doi: 10.1371/journal.pone.0072509 (PMC3738575; doi:10.1371/journal.pone.0072509)
Supplement: Table S5 — Comparison of introduced and native species’ recruitment success once the effect of life form (woody/non-woody) has been taken into account. (DOC) [file pone.0072509.s005.doc]

**Table S5:** Comparison of introduced and native species’ recruitment success once the effect of life form (woody/non-woody) has been taken into account.

**1) PROPORTION OF NATIVE AND INTRODUCED SPECIES WITH WOODY/NON-WOODY LIFE FORM**

|  | **Species’ status** | |
| --- | --- | --- |
| **Life-form** | **Introduced species** | **Native species** |
| Non-woody | 42 (66.7 %) | 118 (41.7 %) |
| Woody | 21 (33.3 %) | 165 (58.3 %) |

**2) SURVIVAL THROUGH GERMINATION AND LIFE FORM**

| **Terms** | **Estimate** | **Standard error** | ***P*** |
| --- | --- | --- | --- |
| Intercept | -2.48 | 0.39 | < 0.0001 |
| Species' status | 0.32 | 0.45 | 0.47 |
| Life form | 0.45 | 0.62 | 0.47 |
| Species' status × Life form | -0.14 | 0.68 | 0.84 |

**3) EARLY SEEDLING SURVIVAL (ONE WEEK AFTER GERMIANTION) AND LIFE FORM**

| **Terms** | **Estimate** | **Standard error** | ***P*** |
| --- | --- | --- | --- |
| Intercept | 2.29 | 0.35 | < 0.0001 |
| Species' status | 0.17 | 0.46 | 0.72 |
| Life form | 1.57 | 0.68 | 0.02 |
| Species' status × Life form | -0.97 | 0.77 | 0.21 |

**4) SURVIVAL FROM GERMINATION TO FIRST REPRODUCTION AND LIFE FORM**

| **Terms** | **Estimate** | **Standard error** | ***P*** |
| --- | --- | --- | --- |
| Intercept | -1.59 | 0.71 | 0.03 |
| Species' status | -0.66 | 0.94 | 0.48 |
| Life form | -0.74 | 1.17 | 0.53 |
| Species' status × Life form | -0.94 | 1.58 | 0.56 |
