# Supplementary material for: A Comparison of the Recruitment Success of Introduced and Native Species Under Natural Conditions
Source: PLoS One. 2013 Aug 8;8(8):e72509. doi: 10.1371/journal.pone.0072509 (PMC3738575; doi:10.1371/journal.pone.0072509)
Supplement: Table S6 — Comparison of introduced and native species’ recruitment success once the effect of continuous longevity has been accounted for. (DOC) [file pone.0072509.s006.doc]

**Table S6:** Comparison of introduced and native species’ recruitment success once the effect of continuous longevity has been accounted for.

We collected maximum recorded longevity (continuous longevity) data from the global literature. Data were collected from a search performed in ISI web of knowledge using the search string: (“longevity” or “lifespan” or “maximum age”) and (“tree” or “shrub” or “herb” or “plant” or “grass”) not (“leaf lifespan” or “floral longevity” or “seed longevity”), only in fields (“plant sciences”, “environmental sciences”, “ecology”, “forestry”, “evolutionary biology”, “developmental biology” and “demography”). Only articles or reviews in English were considered. The search was performed the 5^th^ of December of 2012 and contained information for papers published after 2001. Information on maximum longevity data from papers published before 2001 were sourced from Moles et al. . For 17 species the data on maximum longevity was extracted from Ramula et al. . Finally, the maximum longevity of three species was estimated using the species’ population matrices (following ). First, for each species, the fecundity values from the population matrices were set to zero. Then a population vector with one seedling and zero adults was multiplied by the population matrices until the summed probability of survival for all size classes summed up to 0.01. In total we were able to collect longevity data for 128 species, 23 introduced and 105 native. All longevity data were log_10_-transformed before analysis.

**1) SURVIVAL THROUGH GERMINATION AND CONTINUOUS LONGEVITY**

| **Terms** | **Sum of squares** | **d.f.** | ***P*** |
| --- | --- | --- | --- |
| Intercept | 65.28 | 1 | 0.0004 |
| Species' status | 16.32 | 1 | 0.07 |
| Continuous longevity | 15.77 | 1 | 0.07 |
| Species' status × Continuous longevity | 19.42 | 1 | 0.05 |
| Residuals | 471.33 | 97 |  |

**2) EARLY SEEDLING SURVIVAL (ONE WEEK AFTER GERMINATION) AND CONTINUOUS LONGEVITY**

| **Terms** | **Sum of squares** | **d.f.** | ***P*** |
| --- | --- | --- | --- |
| Intercept | 72.99 | 1 | < 0.0001 |
| Species' status | 3.82 | 1 | 0.25 |
| Longevity | 5.35 | 1 | 0.18 |
| Species' status × Continuous longevity | 0.414 | 1 | 0.71 |
| Residuals | 146.789 | 51 |  |

**3) EARLY SEEDLING SURVIVAL (ONE WEEK AFTER GERMINATION), SEED MASS, AND CONTINUOUS LONGEVITY**

| **Terms** | **Sum of squares** | **d.f.** | ***P*** |
| --- | --- | --- | --- |
| Intecept | 65.14 | 1 | <0.0001 |
| Species' status | 6.95 | 1 | 0.16 |
| Continuous longevity | 2.22 | 1 | 0.42 |
| Seed mass | 2.82 | 1 | 0.36 |
| Species' status × Continuous longevity | 0.03 | 1 | 0.93 |
| Species' status × Seed mass | 1.18 | 1 | 0.56 |
| Continuous longevity × Seed mass | 0.22 | 1 | 0.8 |
| Species' status × Continuous longevity × Seed mass | 0.69 | 1 | 0.65 |
| Residuals | 140.92 | 42 |  |

**4) SURVIVAL FROM GERMINATION TO FIRST REPRODUCTION AND CONTINUOUS LONGEVITY**

| **Terms** | **Sum of squares** | **d.f.** | ***P*** |
| --- | --- | --- | --- |
| Intercept | 25.99 | 1 | 0.04 |
| Species' status | 1.15 | 1 | 0.65 |
| Continuous longevity | 0.7 | 1 | 0.73 |
| Species' status × Continuous longevity | 9.27 | 1 | 0.21 |
| Residuals | 157.15 | 28 |  |

**References**

1. Moles AT, Falster DS, Leishman MR, Westoby M (2004) Small-seeded species produce more seeds per square metre of canopy per year , but not per individual per lifetime. J Ecol 92: 384-396.

2. Ramula S, Knight TM, Burns JH, Buckley YM (2008) General guidelines for invasive plant management based on comparative demography of invasive and native plant populations. J Appl Ecol 45: 1124-1133.

3. Caswell H (2001) Matrix population models: construction, analysis, and interpretation. Sunderland, MA, USA: Sinauer. 722-722 p.

4. Forbis TA, Doak DF (2004) Seedling establishment and life history trade-offs in alpine plants. Am J Bot 91: 1147-1153.
